# Dyspnea Caused by Atlantoaxial Subluxation in a Patient with Rheumatoid Arthritis

**DOI:** 10.1155/2012/170956

**Published:** 2012-02-01

**Authors:** Hsin-Yi Lin, Chien-Chih Chen, Yi-Kung Lee, Yung-Cheng Su

**Affiliations:** ^1^Emergency Department, Buddhist Tzu Chi Dalin General Hospital, no. 2, Minsheng Road, Dalin Township, Chiayi County 622, Taiwan; ^2^Division of Emergency Medicine, Keelung Hospital, Department of Health, Executive Yuan, Keelung 201, Taiwan; ^3^School of Medicine, Tzu Chi University, Hualien 970, Taiwan

## Abstract

Atlantoaxial subluxation is a well-known but poorly recognized disease in rheumatoid patients. We report a patient with rheumatoid arthritis whose chief complaint was dyspnea on arrival to the emergency department (ED). After further investigation, spinal cord compression caused by atlantoaxial subluxation was diagnosed. This is an uncommon but important case that ED physicians should be aware of.

## 1. Introduction

Dyspnea is a possible life-threatening condition that is encountered frequently in the emergency department (ED). We present a patient with rheumatoid arthritis who complained of intermittent shortness of breath. Atlantoaxial subluxation with cervical spinal cord compression was diagnosed after our survey. Because of the timely diagnosis and management, the adverse outcome was prevented.

## 2. Case Report

A sixty-year-old female presented with intermittent shortness of breath happened in the morning of the day. She was a patient with chronic rheumatoid arthritis (RA) and was regularly followed in the rheumatologic clinic. During the past two weeks, she also complained of progressive numbness over bilateral hands and feet. Since the symptoms were not resolved by her daily medications and became more severe in the evening, she was brought to the ED for further evaluation.

At the ED, the patient was ambulatory, cooperative, and in mild distress, complaining of shortness of breath. Her vital signs were temperature 36.8°C, pulse rate 110 beats/min, respiratory rate 24 breaths/min, blood pressure of 168/81, and pulse oximetry 97%. Breathing sounds were normal without crackles or wheeze while auscultation. Other physical examination was unremarkable. Arterial blood gas was obtained and the data were within normal range. Because of the elevated D-dimer level, computed tomography of the chest was arranged to rule out the pulmonary embolism, but there was no significant finding.

**Figure 1:**
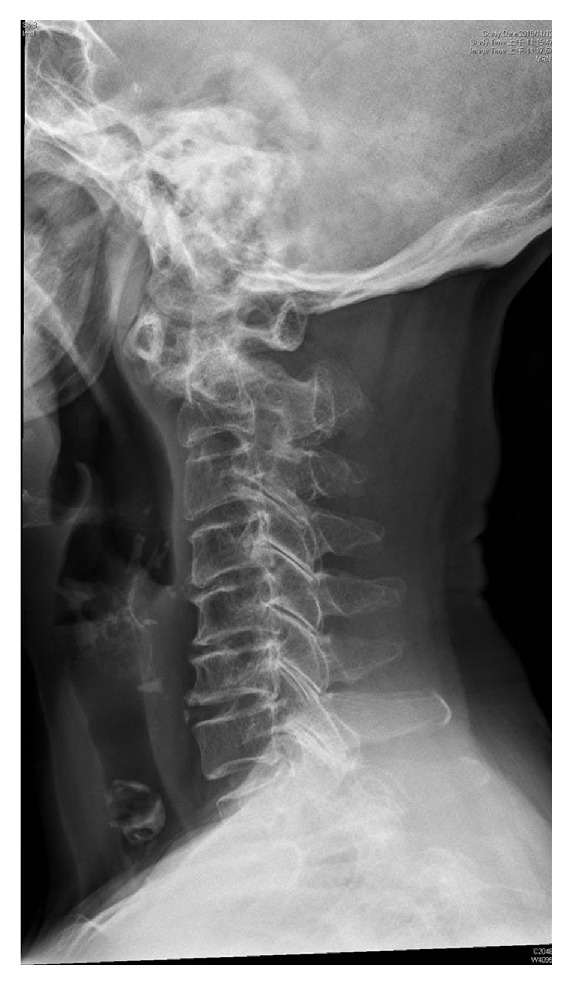
Increased distance at atlantoaxial space was noted.

The patient felt better after the use of oxygen during observation. However, reviewing of the medical records indicated the X-ray of the cervical spine was taken one week ago for the evaluation of limb numbness, and the increased distance at atlantoaxial space was noted (Figure 1). Magnetic resonance imaging of the cervical spine was arranged and atlantoaxial subluxation with acute cervical spinal cord compression over C1-C2 level was found. The neurosurgeon was consulted and surgical intervention was suggested. However, the patient refused operation and conservative treatments with neck immobilization, and steroids were initiated during hospitalization. The patient was discharged 5 days thereafter with preserved neurologic function.

## 3. Discussion

Dyspnea is commonly observed in ED patients. Most of the critical causes are of pulmonary and cardiac origin, such as pulmonary embolism and congestive heart failure, leading many ED physicians *to* overlook other possible important causes. In our patient, the presentation could be nonspecific and led physicians to the wrong diagnosis. However, the underlying condition of the rheumatoid patient made us take spinal lesion into consideration. Atlantoaxial subluxation was noted and spinal cord compression was diagnosed. The patient was then appropriately treated accordingly.

Atlantoaxial subluxation is a well-known yet poorly recognized condition in patients with rheumatoid arthritis. The possible pathogenesis might be the progress of the inflammatory process from adjacent joints or the apophyseal joint destruction [1]. Physical findings and characteristics of the disease are variable. Most patients had neck pain, sensory deficits over hands over feet, and eventually spastic quadriparesis. Respiratory dysfunction can be present [2] but rarely is the main complaint, making the diagnosis in our patient difficult. A plain radiographic view of the cervical spine is important in ruling in the diagnosis. More than 3 mm of distance between the odontoid peg and the C1 indicates possible atlantoaxial subluxation [3], and further magnetic resonance imaging can confirm the diagnosis and evaluate the extent of spinal cord compression. Once atlantoaxial subluxation is suspected, stabilization of the cervical spine and consultation of the neurosurgeon are necessary for further intervention.

In conclusion, emergency physicians should be aware of dyspnea caused by atlantoaxial subluxation, especially in the patients with rheumatoid arthritis. A high index of suspicion and prompt management should avoid adverse outcomes.
